# Calcitonin gene related peptide α is dispensable for many danger-related motivational responses

**DOI:** 10.1038/s41598-021-95670-8

**Published:** 2021-08-10

**Authors:** Joanna Zajdel, Johan Sköld, Maarit Jaarola, Anand Kumar Singh, David Engblom

**Affiliations:** Center of Social and Affective Neuroscience, Department of Biomedical and Clinical Sciences, Linköping University, 58185 Linköping, Sweden

**Keywords:** Feeding behaviour, Amygdala

## Abstract

Calcitonin gene related peptide (CGRP) expressing neurons in the parabrachial nucleus have been shown to encode danger. Through projections to the amygdala and other forebrain structures, they regulate food intake and trigger adaptive behaviors in response to threats like inflammation, intoxication, tumors and pain. Despite the fact that this danger-encoding neuronal population has been defined based on its CGRP expression, it is not clear if CGRP is critical for its function. It is also not clear if CGRP in other neuronal structures is involved in danger-encoding. To examine the role of CGRP in danger-related motivational responses, we used male and female mice lacking αCGRP, which is the main form of CGRP in the brain. These mice had no, or only very weak, CGRP expression. Despite this, they did not behave differently compared to wildtype mice when they were tested for a battery of danger-related responses known to be mediated by CGRP neurons in the parabrachial nucleus. Mice lacking αCGRP and wildtype mice showed similar inflammation-induced anorexia, conditioned taste aversion, aversion to thermal pain and pain-induced escape behavior, although it should be pointed out that the study was not powered to detect any possible differences that were minor or sex-specific. Collectively, our findings suggest that αCGRP is not necessary for many threat-related responses, including some that are known to be mediated by CGRP neurons in the parabrachial nucleus.

## Introduction

Calcitonin gene related peptide-expressing neurons in the external lateral part of the parabrachial nucleus (CGRP^PBN^ neurons) have been proposed to serve as a universal danger sensor. They are activated by a wide variety of noxious stimuli^1,2^, ranging from painful electric shocks, pinching and heat pain^1,3^, to malaise-inducing LiCl, lipopolysaccharide (LPS), satiety hormones^4,5^ and malignancies^6^. Activation of CGRP^PBN^ neurons enables appropriate behavioral reactions to danger, such as suppression of appetite in conditions when food consumption might be harmful^4,7^, scratching in response to itch^1^ and the generation of threat memories^3^.

CGRP^PBN^ neurons send projections to the central amygdala (CeA) and other forebrain structures^8^. The projection to the extended amygdala have been shown to be important for several pain-related responses^3,9^. While the role of CGRP^PBN^ neurons is well established, less is known about the involvement of CGRP in the processes they mediate. Injections of CGRP to the amygdala have been reported to facilitate both synaptic transmission and pain responses^10^, but also to have an antinociceptive effect^11^, while infusions of CGRP receptor 1 antagonists have been demonstrated to block synaptic plasticity accompanying the development of chronic pain in rat model of arthritis^12^. Further, CGRP has been shown to modulate conditioned taste aversion induced by LiCl but not aversion induced by activation of CGRP^PBN^ neurons^13^.

To address the role of αCGRP in threat-related responses, including such that are known to be mediated by CGRP^PBN^ neurons, we used a transgenic mice with deletion of *Calca*^14^, the gene encoding αCGRP, which is the primary form of CGRP in the brain. In these mice, we investigated a broad panel of threat-related responses and behaviors known to be mediated by CGRP^PBN^ neurons including food intake suppression and pain-related responses.

## Materials and methods

### Animals

All experiments were approved by the Linköping Animal Care and Use Committee and followed national and international guidelines including ARRIVE. Unless otherwise specified, mice were kept on a 12 h light/dark cycle with ad libitum access to food and water, in a room with controlled humidity and temperature. Pain, palatable food intake and c-fos induction experiments were performed during the light phase while conditioned taste aversion and anorexia experiments were performed during the dark phase. Animals were of both sexes and at least 7 weeks old at the start of the experiments. αCGRP-KO mice^14^ were obtained from MMRRC (RRID:MMRRC_036773-UNC) and maintained on C57BL/6 background. In these mice a membrane-tethered axonal tracer (farnesylated enhanced GFP) flanked by loxP-sites followed by a coding sequence for the human diphtheria toxin receptor was introduced into the *Calca* locus. The cassette disrupts the expression of *Calca* and instead GFP is expressed in Calca-positive cells. If Cre is expressed in the cells (this was never the case in this study) the cells instead express the human diphtheria toxin receptor. The transgenic line was originally generated for visualization and conditional ablation of *Calca* expressing cells^14^, but without administration of diphtheria toxin it can be used as a *Calca*-KO and a way to visualize *Calca*-positive cells. Mice homozygous for the transgene are referred to as αCGRP-KO, heterozygous mice as HZ and mice without the transgene as WT. In behavioral experiments KO and WT animals were used. In histological experiments, WT were sometimes replaced by HZ since it is possible to visualize the CGRP-cells with GFP in the latter. All genotypes were bred in house as one line. Experimental animals were littermates except for a very few animals that were first generation of offspring from littermates. In the immunohistochemical experiment involving only WT mice, mice from a different line were used.

### C-fos induction and immunofluorescence

Mice were perfused with saline followed by 4% paraformaldehyde in PBS. The brains were postfixed in the same solution for 4 h in 4 °C and placed in 30% sucrose till saturated. For c-fos induction experiments, mice were injected with LPS (10 µg/kg, *E. coli*, 055:B5, Sigma, dissolved in sterile saline) or saline 3 h before perfusion. 40 µm sections were cut in a cryostat and later blocked in 10% Normal Donkey Serum in PBST (PBS and 0.4% Triton-X) for 24 h. After blocking, sections were incubated in a mixture of primary antibodies (rabbit anti-CGRP, 1: 5000, PenLab, T-4031 or rabbit anti-C-fos, 1:1000, Abcam, 190289; chicken anti-GFP, 1:10,000, Abcam, ab13970) over night and later in mixture of secondary antibodies (goat anti-chicken Alexa Fluor 488, 1:1000 and donkey anti-rabbit Alexa Fluor 568, 1:1000 (CGRP staining) or 1:200 (c-fos staining)) for 2 h. For double staining of CGRP and c-fos, sections were blocked for 45 min in 4% bovine serum albumin and 0.2% Triton-X, incubated in rabbit anti-C-fos overnight followed by 2 h of incubation in donkey anti-rabbit Alexa Fluor 568. They were then blocked in 10% normal donkey serum in PBST and incubated in rabbit anti-CGRP overnight followed by 2 h incubation in donkey anti-rabbit Alexa Fluor 488, 1:1000. For c-fos quantification, the external lateral part of the PBN was identified with the help of CGRP/GFP immunoreactivity. Pictures of the PBN were taken with a Leica DMi8 fluorescent microscope. Images were processed with Fiji^15^ by a researcher blinded to the treatment. Background was subtracted using the built-in rolling ball algorithm. A threshold was set and the number of c-fos positive cells in the PBN were counted with the “Analyze particles” function. All other images were taken with a Zeiss LSM700 upright confocal microscope. For the histology experiments we used 8 WT mice (4 males and 4 females), 8 HZ mice (4 males and 4 females) and 4 KO mice (2 males and 2 females).

### Quantitative PCR

Mice were made unconscious with CO2 and decapitated. The brain stem was dissected and stored in RNAlater. RNA was extracted using RNeasy Lipid Tissue Kit (Qiagen) according to instructions from the manufacturer. Synthesis of cDNA was performed with High Capacity cDNA Reverse Transcription Kit (Applied Biosystems) and quantitative PCR was performed with TaqMan assays (Calca: Mm 00801463_g1, Gapdh: Mm99999915_g1), according to the instructions from the provider. Reaction was performed in a Real-Time 7500 Fast apparatus (Applied Biosystems) and quantification was done with the ΔΔCT method^16^ with GAPDH as control gene. For the qPCR experiments we used 3 WT mice (1 male and 2 females) and 4 KO mice (3 males and 1 females).

### Behavioral testing: general procedures

In the behavioral tests, the experimenters were blind to the genotype of the animals. A separate cohort of mice was used for each test if not otherwise stated. We used littermates in the different experimental groups. In very rare cases we instead used the first generation of offspring from littermates.

### LPS-induced anorexia

Mice were single-housed 5 days before the start of the experiment. One hour before the onset of the dark phase, animals were injected with saline or LPS i.p. (10 µg/kg, *E. coli*, O111:B4, Sigma, dissolved in sterile saline) and chow was withdrawn. At the onset of the dark phase, the mice got access to food and the intake was measured 3 and 6 h later. Male mice were used in this experiment.

### Conditioned taste aversion induced by LPS, and neophobia

Mice were single housed and moved to a room with inverted light cycle (lights on at 11 pm) 2 weeks before the experiment. Animals were habituated to 4 h water deprivation (from the onset of dark phase) for 4 days. On the conditioning day, following 4 h water deprivation, mice got access to 0.15% (w/v) saccharin in tap water. After 1 h, mice received LPS i.p. (10 µg/kg, *E. coli*, O111:B4, Sigma, dissolved in saline) or saline, and the baseline intake of saccharin was measured. During the next 2 days, animals were exposed to 4 h water deprivation. The third day after LPS treatment, following 4 h water deprivation, animals got access to saccharine solution for 1 h and the intake was measured. Mice drinking less than 0.5 g of saccharine solution during the conditioning day were excluded in the second measurement, since in our experience consuming less than this amount is not enough to elicit reliable taste avoidance. The results shown come from two separate cohorts. Neophobia was assessed based on measurements obtained during the conditioned taste aversion experiments. For the analysis we used the saccharin intake from the conditioning day of CTA, before saline or LPS injections. All animals were included (i.e. both saline and LPS groups). This intake was compared to the intake 3 days later and here we included data only from the mice which had been previously injected with saline. In this experiment we used 20 WT mice (14 females and 6 males) and 20 mice lacking αCGRP (14 females and 6 males).

### Palatable food intake

Mice were single-housed 5 days before the start of the experiment. Consumption of Fresubin Original drink, chocolate flavour, was measured for 2 h, after a pre-exposition to the flavour. In this experiment we used 10 WT mice (6 males and 4 females) and 11 KO mice (9 males and 2 females).

### Formalin test

Animals were placed in a transparent plexiglass box 20 (w) × 15 (d) × 25 (h) cm for 30 min. After the acclimatization period, 20 µl of diluted formalin (2.5%) was injected below the skin on dorsal side of the right hindpaw. The behavior was recorded for 60 min. Time spent performing nociceptive behaviors (paw licking, biting and shaking) was measured. A nociceptive score was calculated that corresponds to the share of time used performing nociceptive behaviors for each 5 min timeslot. Male mice were used in this experiment.

### Conditioned place aversion induced by thermal pain

Mice were single-housed at least 1 day before start of experiment. An Incremental Hot/Cold Plate Analgesia Meter (IITC Inc Life Science) was modified to serve as a conditioned place preference box with two chambers. On day 1 (pre-test), animals explored the apparatus for 15 min. If the time spent in one chamber exceeded 12 min, the mouse was excluded. On days 2–5 animals were placed in a non-preferred chamber with the temperature set to 30 °C, and in the preferred chamber with the temperature set to 45 °C. The conditioning sessions lasted 10 min or until the mouse jumped three times. They were separated by at least 4 h and the order of the sessions was altered daily. On day 6, post-test was performed the same way as the pre-test. The aversion scores were calculated as the difference between the times spent in the pain-paired chamber in the post-test minus the times spent in the same chamber in the pre-test. Male mice were used in this experiment.

### Heat escape test

Mice were single-housed at least 1 day before start of experiment. A 12 cm high platform was placed inside a plexiglass enclosure on an Incremental Hot/Cold Plate Analgesia Meter (IITC Inc Life Science). The floor temperature was set to 45 °C. The animals were placed on the hot floor and the time until they jumped, or tried to jump, up onto the platform was measured. Only male mice were included in the experiment since, according to our experience, many female mice do not climb the platform (most likely this is due to the fact that they are smaller and the platform is more difficult to climb for them) leading to high variability within the groups.

### Acetic acid locomotion

On day 1, mice previously tested in the heat escape test were habituated to an open field box (44 × 44 cm). After 20 min they were injected with 100 µl saline intraperitoneally. The same procedure was repeated on day 2 and the session was recorded. On day 3 the saline was replaced with 0.6% acetic acid dissolved in saline. Videos were analyzed with Ethovision XT tracking software (Noldus) to measure locomotion. Reduction in locomotion was calculated as distance moved after saline injection subtracted from distance moved after acetic acid injection. For this experiment 9 WT mice (4 males and 5 females) and 11 KO mice (5 males and 6 females) were used.

### Statistical analysis

Data were analyzed with GraphPad Prism. Results are presented as mean ± SEM and individual values are indicated by dots. Kolmogorov–Smirnov and Shapiro–Wilk tests for normality indicated that all behavioral data had an approximately normal distribution, except for the anorexia data that were approximately lognormal. Accordingly, parametric tests were used for all data and for the anorexia experiment statistical tests were done on log transformed data. Comparisons of two groups were done using two-tailed Student’s T-test and comparisons involving more than two groups were performed using one-way or two-way ANOVA followed by Holm–Sidak's multiple comparisons test. *p* values < 0.05 were considered statistically significant.

## Results

### Loss of CGRP in the projections to the central amygdala in αCGRP-KO mice

**Figure 1:**
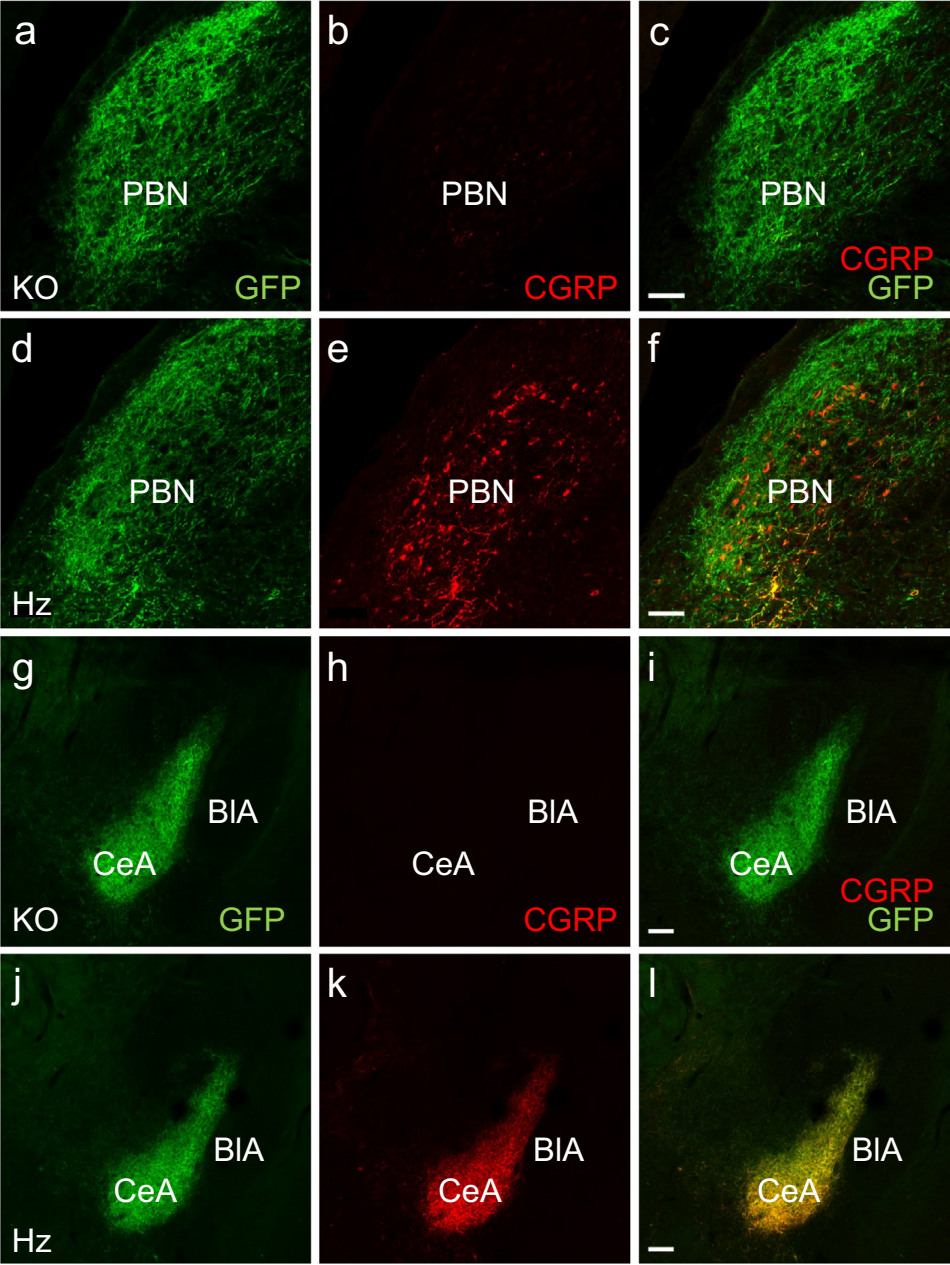
CGRP levels are strongly attenuated in αCGRP-KO mice. (**a**–**f**) Micrographs from confocal microscopy showing that the CGRP-immunoreactivity in the parabrachial nucleus was strongly reduced in the αCGRP-KO mice compared to heterozygous (Hz) mice. Scale bar 50: µm. (**g**–**l**) CGRP-immunoreactivity was present in the central nucleus of the amygdala of Hz, but not αCGRP-KO mice. Scale bar: 100 µm. *PBN* parabrachial nucleus, *CeA* central amygdala, *BlA* basolateral amygdala.

To confirm the deletion of CGRP in the parabrachial-amygdaloid pathway, we compared the CGRP expression in αCGRP-KO animals carrying two copies of the transgene (farnesylated eGFP knocked-in to the *Calca* locus) to that of heterozygous mice (carrying one copy of the transgene and one intact *Calca* allele). In both genotypes, GFP was found in the cell membranes of PBN neurons and their projections to the amygdala. GFP-immunoreactivity was used to facilitate identification of these structures in the αCGRP-KO mice. We found only very weak CGRP-immunoreactivity in the PBN of αCGRP-KO mice whereas strong CGRP labeling was seen in heterozygous mice (Fig. 1a–f). Furthermore, no CGRP immunorectivity was detected in the CeA of αCGRP-KO mice (Fig. 1g–l), indicating a loss or a very strong reduction of CGRP signaling in this area. It was obvious that the CGRP-labeling was low or absent also in other brain structures such as the trigeminal dorsal horn and in motor neurons of cranial nerve nuclei although we did no structured analysis of these structures. In addition, qPCR analysis for *Calca* mRNA in the brainstem showed that αCGRP-KO mice expressed virtually no *Calca* mRNA (WT: 100 ± 1.8%, KO: 0.01 ± 0.01%, *p* = 0.0003, Student’s t-test).

### A low dose of lipopolysacharide activates CGRP-neurons in the parabrachial nucleus

**Figure 2:**
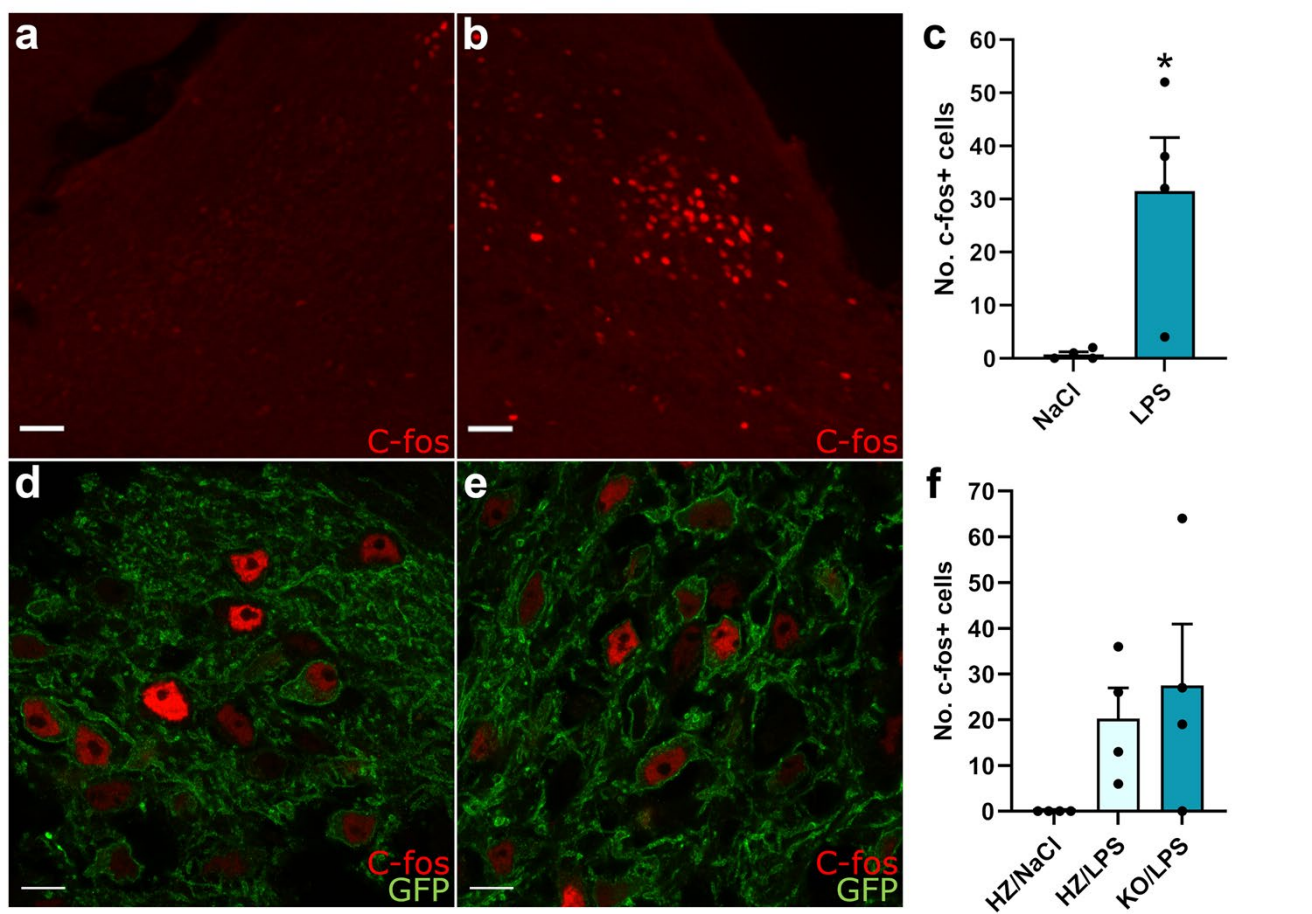
C-fos expression in the PBN after LPS-injection. (**a**,**b**) Representative fluorescent images showing c-fos immunoreactivity in the PBN of one NaCl (**a**) and one LPS (**b**) injected WT animal. Scale bar 50 µm. (**c**) The amount of C-fos positive cells in the PBN is higher 3 h after LPS injection i.p. compared to 3 h after NaCl injection in WT animals (T-test: *p* = 0.02, NaCl n = 4, LPS n = 4). (**d**,**e**) Representative confocal images of the PBN showing c-fos expression (red) in CGRP-neurons expressing GFP (green) from one Hz (**d**) and one KO (**e**) animal. Scale bar 10 µm. (**f**) LPS induced c-fos expression in both Hz and KO mice (Hz/NaCl n = 4, Hz/LPS n = 4, KO/LPS n = 4).

Intraperitoneal injection of LPS has been shown to activate CGRP^PBN^ neurons^4^. We examined if a low dose (10 µg/kg) of LPS activated CGRP^PBN^ neurons in our experimental setting using immunofluorescent detection of the activity marker c-fos. As expected, LPS induced strong activation of neurons in the external lateral part of the PBN (Fig. 2a–c). Closer examination using dual labeling of GFP and c-fos in animals expressing eGFP under the Calca-promotor revealed an extensive co-localization showing that many of the activated neurons were expressing CGRP (Fig. 2d–e). We next analyzed the activation of the PBN in αCGRP-KO mice and found strong c-fos expression in both αCGRP-KO mice and their HZ littermates (Fig. 2f).

### CGRP signaling is not critical for appetite suppression induced by fullness, neophobia or inflammation

**Figure 3:**
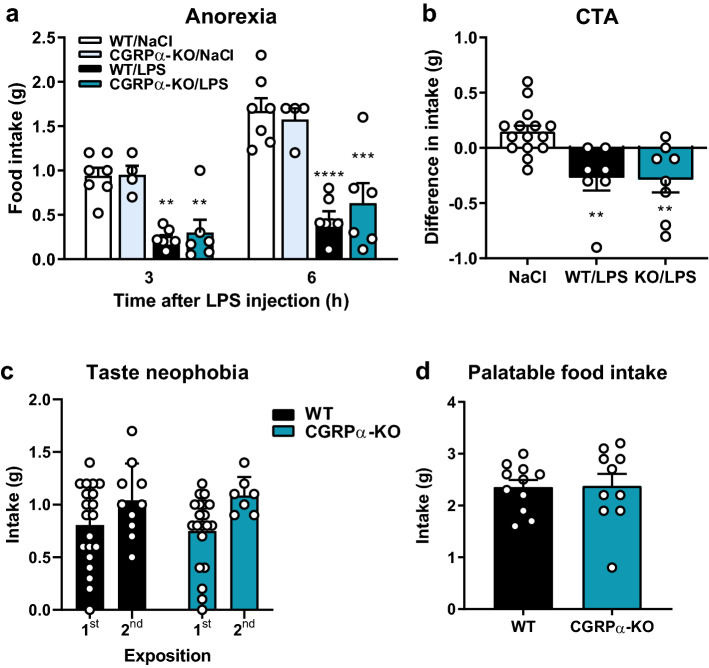
Food consumption in αCGRP-KO mice. (**a**) Inflammation-induced anorexia was similar in αCGRP-KO and WT animals. Two-way ANOVA followed by Holm–Sidak's multiple comparisons test: 3 h: treatment: *F*_(1,20)_ = 35.51, *p* < 0.0001; genotype: *F*_(1,20)_ = 0.0055; *p* = 0.82; interaction: *F*_(1,20)_ = 0.11; *p* = 0,74. 6 h: treatment: *F*_(1,20)_ = 26.81, *p* < 0.0001; genotype: *F*_(1,20)_ = 0.011; *p* = 0.92; interaction: *F*_(1,20)_ = 0.085; *p* = 0,77. Stars indicate comparisons within the same genotype. *n* = 6 in both groups. (**b**) Both WT mice and mice without αCGRP developed conditioned taste avoidance to LPS. One-way ANOVA followed by Holm–Sidak's multiple comparisons test: *F*_(2, 27)_ = 9.605, *p* = 0.0007. NaCl *n* = 8 WT + 7 αCGRP-KO, WT/LPS *n* = 11, αCGRP-KO/LPS *n* = 8. (**c**) αCGRP-KO mice show taste neophobia to a similar degree as WT mice. Two-way ANOVA followed by Holm-Sidak's multiple comparisons test: interaction: *F*_(1,53)_ = 0.24, *p* = 0.625; time: *F*_(1,53)_ = 7.8; *p* = 0.0074; genotype: *F*_(1,53)_ = 0.002; *p* = 0.96. WT 1st *n* = 20, WT 2nd *n* = 10, αCGRP-KO 1st *n* = 20, αCGRP-KO 2nd *n* = 7. (**d**) Palatable food intake was unaffected by deletion of *Calca.* T-test: *p* = 0.92; WT *n* = 11, αCGRP-KO *n* = 10. **p < 0.01; ***p < 0.001; ****p < 0.0001.

We next examined a number of responses related to threat-induced regulation of food-intake in αCGRP-KO mice. As CGRP^PBN^ neurons have been shown to be important for the reduction of food intake induced by inflammation^4^, we assessed if CGRP is important for limitation of food intake under inflammatory conditions. αCGRP-KO mice developed an inflammation-induced anorexia that was similar to that of WT mice, as measured by consumption of standard chow 3 and 6 h after an ip. injection of LPS (10 µg/kg; Fig. 3a). CGRP^PBN^ neurons are also important for conditioned taste avoidance, i.e. the ability to avoid taste stimuli after their association with nausea or malaise^17^. We exposed animals to a novel taste (0.15% saccharin solution) and administered LPS (10 µg/kg) afterwards. Both αCGRP*-*KO mice and their WT littermates reduced their saccharin solution intake after the pairing of the sweet taste with inflammation-induced malaise 2 days earlier (Fig. 3b). The WT and αCGRP-KO mice also displayed the same degree of neophobia to the new taste as they drank similar amounts of saccharine solution at the first presentation (Fig. 3c) and this amount was lower than the amount consumed at the second exposure (only values from the NaCl treated group included). CGRP^PBN^ neurons are also important for the prevention of overeating^7^, as measured by meal size. We tested if αCGRP-KO mice with ad libitum access to normal chow displayed an increased intake of palatable food when given access to it for a limited period (2 h). The consumption of a sweet, chocolate-flavored drink was not affected by loss of αCGRP (Fig. 3d).

### Calca deletion does not influence nociceptive behaviors or the affective component of pain

**Figure 4:**
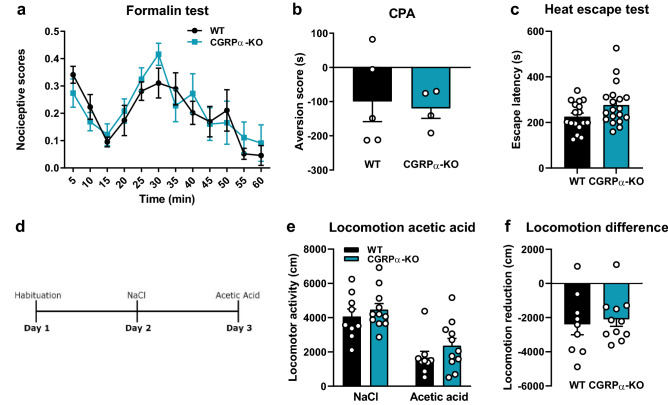
Nociceptive behaviors in αCGRP-KO mice. (**a**) WT and αCGRP-KO mice showed similar responses to inflammatory pain in the formalin test. One-way RM ANOVA: time: *F*_(11, 120)_ = 6.52, *p* < 0.0001; genotype: *F*_(1, 120)_ = 0.351, *p* = 0.555; interaction: *F*_(11, 120)_ = 0.638, *p* = 0.793; *n* = 6 in both groups. (**b**) Conditioned place aversion to thermal pain was similar in αCGRP-KO and WT mice. T-test: *p* = 0.79; WT *n* = 5, αCGRP-KO *n* = 4. (**c**) No statistically significant difference in escape latency was seen between the genotypes in the heat escape test. T-test: *p* = 0.06; WT *n* = 17; αCGRP-KO *n* = 19. (**d**) Timeline of the acetic acid locomotion test. (**e**) No genotype difference was seen in locomotor activity after intraperitoneal injection of saline or 0.6% acetic acid. (**f**) αCGRP-KO and WT mice show similar reduction in locomotion following acetic acid injection compared to after saline injection. WT *n* = 9; αCGRP-KO *n* = 11.

To examine the role of CGRP in pain-related responses, we first injected diluted formalin into the paw of αCGRP-KO mice and their WT littermates and monitored their response to the inflammatory pain for 1 h. αCGRP-KO mice showed nociceptive responses similar to those of WT mice (Fig. 4a). To assess the affective component of pain, we used conditioned place aversion. This is a well-established model for the affective component of pain and measures the avoidance of an environment in which the mice have experienced pain. Both WT and αCGRP-KO mice learned to avoid a chamber where they were subjected to thermal pain (floor with temperature set to 45 °C) (Fig. 4b). αCGRP-KO tended to stay longer on the hot floor in the heat escape test (Fig. 4c), but the difference did not reach statistical significance (*p* = 0.06). αCGRP-KO mice showed a similar reduction in locomotion as WT-mice after intraperitoneal injection of 0.6% acetic acid (Fig. 4d–f).

## Discussion

Our findings suggest that αCGRP is dispensable for many behaviors mediated by CGRP^PBN^ neurons. This conclusion is based on a comparison of the results from this study, in which we use an intervention against CGRP, to the results from studies manipulating the activity of CGRP^PBN^ neurons. To pick a few examples, inhibition of the CGRP^PBN^ neurons diminished LPS-induced anorexia^4^ and LPS-induced conditioned taste aversion^17^. We show that these behavioral responses are unaffected by the lack of αCGRP in the parabrachial-amygdaloid pathway. Further, our results indicate that *Calca* deletion does not lead to overeating of palatable food in sated mice nor excessive intake of a liquid with a novel taste, even if the CGRP^PBN^ neurons have been shown to be important for protection from overfeeding^7^ and for taste neophobia^1^. Along the same line, functional silencing of CGRP^PBN^ neurons by tetanus toxin impairs the fear response associated with painful foot-shocks^3^ but we demonstrate that αCGRP-KO mice do not differ from WT mice in their affective responses to thermal pain, since they avoid the context associated with nociceptive stimulation. CGRP^PBN^ neurons have also been shown to mediate escape behavior (jumping) in the hot plate test^3^. We find that αCGRP KO-mice tend to stay longer on a hot floor in the heat escape test. However, the difference was not statistically significant (*p* = 0.06) even though many animals were tested, indicating that the possible role of CGRP is minor. It has previously been shown that αCGRP is important for conditioned taste aversion induced by LiCl but not conditioned taste aversion induced by activation of CGRP^PBN^ neurons^13^. Our finding, that conditioned taste aversion induced by LPS is not dependent on αCGRP, is not directly contradictory to these findings and together they may indicate that CGRP is important for some, but not most, of the danger related responses induced by CGRP^PBN^ neurons.

Our results indicate that even if *Calca* can be used as a marker for a specific subpopulation of parabrachial neurons encoding danger signals, the αCGRP itself is not necessary for many aspects of such encoding. Instead, it is likely that the release of glutamate by the same neurons^18^ is sufficient to mediate the responses studied. In general, it is not surprising that the molecules we use as markers for different cell population do not mediate all functions of these populations. Accordingly, similar disconnects between the function of the marker and the population have been reported for other neuropeptides. For example, deletion of Agouti-Related Protein has no^19^ or only minor^20^ effects on food-intake and energy homeostasis, which contrasts to the strong effects seen after activation, inactivation^21,22^ or ablation of the hypothalamic neurons expressing this neuropeptide^23,24^. Even if we found no effect of αCGRP deletion in our experiments, we cannot exclude the possibility that CGRPα in PBN neurons is involved in other aspects of danger-related signaling. Such aspects could include domains not investigated here (e.g. itch-related responses), specific responses not tested but within the domains investigated (e.g. conditioned taste aversion induced by agents other than LPS) and responses to chronic rather than acute challenges.

The fact that mice without αCGRP behave like normal mice in the danger-related tests used here shows that CGRP in structures other than the PBN is also dispensable for these readouts. This is perhaps not surprising at first sight given that we primarily selected tests that are known to be dependent on the activation of parabrachial neurons. On the other hand, it should be pointed out, that αCGRP is found in a large population of primary afferents^25^ and several of the behavioral responses tested in this study are dependent on primary afferents. Nevertheless, we found no obvious differences between wt mice and mice lacking αCGRP in the pain-related responses studied. This might be explained by the fact that the study was not designed to identify subtle effects of CGRP on spinal processing and consequently it is likely that we would have found a role of CGRP if we used a larger battery of tests specifically aim at investigating this. Also, our study was not powered to detect very subtle or sex-specific genotype effects. Our results contrast to a previous study suggesting αCGRP in TRPV1-expressing primary afferents to be important for visceral nociceptive signaling^25^. The reasons for this discrepancy require further studies.

Since we find very little differences between wildtype mice and mice lacking αCGRP, it is important to consider possible compensatory mechanisms in the mouse line used. To start with, the weak CGRP-IR in the PBN can be explained by low expression of a second CGRP isoform, CGRPβ, encoded by a separate gene, *Calcb*^26^. Since αCGRP and βCGRP differ only by three amino acids in mice^27^, they most likely serve similar functions and they are very difficult to distinguish with antibody-based detection methods. Co-expression of *Calca* and *Calcb* has been reported in different cranial nerve nuclei^26^. However, no CGRP-IR was detected in the projections to the CeA in our mice lacking *Calca*, which indicates that CGRP signaling in this structure was absent or reduced to negligible levels, although the immunofluorescent labeling used here does not allow an exact quantitative assessment. Since αCGRP was lost also during development in these mice, other compensatory mechanisms might have been activated. However, we have previously shown that the mouse line used have deficits in an animal model for hot flushes^28^. This shows that there is no full and general compensation for the lack of αCGRP in this mouse line, but a lack of compensation regarding one readout does not exclude compensation in another response. To comment on another aspect of specificity, it is also unlikely that the co-occurring deletion of calcitonin, also encoded by *Calca*^29^, would mask any effect on behaviors related to nociception and food consumption, since calcitonin is mostly important for calcium homeostasis^30^. Another potential weakness with the mouse line used is that CGRP is deleted in the entire organism. This means that if we would have seen a behavioral difference between the genotypes, we would not have known if it was a due to an effect in the PBN, the spinal cord or elsewhere. However, since we found no effects of the deletion, the fact that it affected the entire organism is instead a strength since it excludes the risk of not affecting all relevant cells.

In summary, we demonstrate that αCGRP is dispensable for many danger-related behaviors, including many that previously have been shown to be mediated by CGRP^PBN^ neurons. This finding suggests that interventions specifically targeting CGRP signaling might be a difficult approach for treating aversion-related symptoms.
